# Validity analysis on merged and averaged data using within and between analysis: focus on effect of qualitative social capital on self-rated health

**DOI:** 10.4178/epih.e2016012

**Published:** 2016-04-08

**Authors:** Sang soo Shin, Young-jeon Shin

**Affiliations:** Department of Preventive Medicine, Hanyang University College of Medicine, Seoul, Korea

**Keywords:** Social capital, Self-assessment, Health status, Multilevel analysis, Korean

## Abstract

**OBJECTIVES:**

With an increasing number of studies highlighting regional social capital (SC) as a determinant of health, many studies are using multi-level analysis with merged and averaged scores of community residents’ survey responses calculated from community SC data. Sufficient examination is required to validate if the merged and averaged data can represent the community. Therefore, this study analyzes the validity of the selected indicators and their applicability in multi-level analysis.

**METHODS:**

Within and between analysis (WABA) was performed after creating community variables using merged and averaged data of community residents’ responses from the 2013 Community Health Survey in Korea, using subjective self-rated health assessment as a dependent variable. Further analysis was performed following the model suggested by WABA result.

**RESULTS:**

Both E-test results (1) and WABA results (2) revealed that single-level analysis needs to be performed using qualitative SC variable with cluster mean centering. Through single-level multivariate regression analysis, qualitative SC with cluster mean centering showed positive effect on self-rated health (0.054, p<0.001), although there was no substantial difference in comparison to analysis using SC variables without cluster mean centering or multi-level analysis.

**CONCLUSIONS:**

As modification in qualitative SC was larger within the community than between communities, we validate that relational analysis of individual self-rated health can be performed within the group, using cluster mean centering. Other tests besides the WABA can be performed in the future to confirm the validity of using community variables and their applicability in multi-level analysis.

## INTRODUCTION

With increasing attention on the effect of environment around individuals as a social decisive factor on health, many multi-level analytic studies are now assessing the simultaneous reflection of health-related occurrence factors at the individual and local community level. Recently, social capital (SC) has gained prominence as a factor affecting individual health. SC, a form of capital developed from social relationship rather than individual relationship, is a concept different from economic, human, or cultural capital [1]. Although there are different viewpoints on defining SC, some quantitative and qualitative factors built from interpersonal relationship affecting SC are reflected in prior studies, including social participation, quality of family relationship, contact frequency, reliability, and reciprocity.

Many studies have confirmed the relationship between SC and health, and recent studies are shifting towards multi-level analysis utilizing the characteristics of SC at the regional level. Two primary data selection methods exist for multi-level analysis of SC reflecting community characteristics. The first involves consideration of administrative data such as turnout of voters, number of volunteer camps, and community-based organizations [2] as proxy variables of SC. The second method utilizes the merged average of individual responses or the response rate. Average participation level in community organizations [2], confidence levels of seven community groups, and individual responses on network level of 11 different types of community organizations were created as community variables [3]. Happiness index calculated by averaging the satisfaction scores of five different sections including individual health and attitude scores as a property of SC, calculated by averaging attitude scores of four different sections including attitude score against disabled people were reflected in the analysis [4].

Although one can ensure objectivity of the data using administrative data as a proxy variable, it is difficult to distinguish whether the data is a result of SC or is a representation of SC. On the contrary, newly created data using individual data allows selection of factors that are closely related to SC compared to administrative data, although potential bias between individuals can result in reduced reliability of the accumulated data. Therefore, one can overcome limitations of the data obtained from administrative data, using survey data through a statistically validated method to represent the community, avoiding unnecessary logical leaps when explaining the relationship between the SC and health. However, a statistical test is needed to validate merging to convert individual responses into community variables. In other words, the procedure to validate whether or not the measurements from combining individual scores are adequately representing the characteristics of the community is necessary [5]. Within and between analysis (WABA) is a method suggested by previous studies to assess construct validity in multi-level analysis. As a tool to assess validity in data merging and adequacy of multi-level analysis, the WABA can provide statistical test results on whether or not the newly calculated community variables can be used as a valid, shared construct, and whether it can be used as an individual variable or must be manipulated into a comparative variable. Domestic studies involving validation of upper-level variables derived from individual variables were often at the organizational level [5-7], and in the domestic public health field, only one study by Lee et al. [8] assessed the validity of utilizing merged and averaged variables from individual self-rated health, stress, and oral health variables, as community variables to discover regional variables affecting the health-related quality of life. There has been no previous study assessing the validity of merged variables from SC and their applicability in multi-level analysis.

Therefore, this study aims to assess the validity of regional level SC variables calculated through merging and averaging the data from community residents’ responses in reliability and reciprocity section of 2013 Community Health Survey, and applicability in multi-level analysis using subjective self-rated health assessment as a dependent variable through WABA.

## MATERIALS AND METHODS

### Subjects and data

The analysis uses data from the 2013 Community Health Survey. This nation-wide survey was conducted from August to November of 2013, for statistical data to understand the health conditions of community residents and to establish and evaluate health policy based on the evidences from the survey. Around 900 people over 19 years of age selected through stratified sampling were included in health-related surveys at the city, district, and borough-levels of the public health centers. Out of 221,816 surveys, 215,727 surveys were used in the analysis excluding 6,089, which did not have responses for key questions.

### Analyzed variables

Self-rated health question of “What do you think of your usual health conditions?” with very bad, bad, average, good, and very good as potential answers was used as dependent variable. This is a continuous variable where a higher score is assigned to the more positive answer for the question.

The independent variables were reliability and reciprocity. The question to assess reliability was “Can you and your neighbors within the community trust each other?”, and the one to assess reciprocity was “Do people within the community have the tradition to help each other for congratulations and condolences?” with an option to choose “yes” or “no” for both questions. Results were converted into continuous variables by assigning one point for every “yes” answer and zero point for every “no” answer, with the total score ranging from zero to two, and this was termed qualitative SC. Qualitative SC was manipulated into community variables by merging and averaging community residents’ individual responses, and finally the cluster group mean centering variable was calculated by subtracting merged and averaged community scores from individual scores, which was then termed “mean-centered qualitative SC”.

Control variables included sex, age, education level, income, marital status, and contact frequency with family, friends, and neighbors. Sex (male, female), age (19-45, 46-65, 65+), educational level (none, elementary school graduate, middle school graduate, high school graduate, above college attending or graduate), monthly income (Korean won [KRW]; <1, 1-2 million KRW, 2-3 million KRW, ≥4 million KRW, no answer), and marital status (single, married, married but without partner) were all designed as nominal variables. Contact frequency with family, friends, or neighbors changed from frequency variable (>4 times/wk, <3 times/wk to >1 times/mo, <1 times/mo) to nominal variable (frequent, sometimes, rarely).

### Statistical analysis

First, basic statistics were calculated for variables in the analysis to understand the basic characteristics of the data. Next, reliality and reciprocity variables were merged, and the community variable was produced using community residents’ average responses, followed by assessment of Spearman’s rank correlation between control and independent variables. Validity and adequacy analysis was performed through the WABA. The WABA provides not only the validity of merging but also analysis level based on relationship between the variables. The analysis is divided into WABA(1) and WABA(2). WABA(1) provides validity of community variables using results from analysis of variance for individual independent variables, while WABA(2) allows determination of analysis level by looking at differences between and within the group(s) in the relationship between independent and dependent variables [5,8,9].

WABA(1) considers the sizes of between group variation and within group variation, testing using the value of η_B_/η_W_. η_W_ is calculated as the square root of SSW/SST, and η_B_ is calculated as the square root of SSB/SST, and E-test is performed using these values. E-test involves the two vectors (B and W) forming the two axes forming 90° and between and within group deviations changing between the two axes. E values of 1.3 (at 52.5°, +7.5° away from the middle 45°) and 0.77 (at 37.5°, -7.5° away from the middle 45°) are used as test standards. E≥1.3 represents differences between the groups and within group homogeneity, while E≤0.77 represents large differences within the group and relatively smaller differences between the groups suggesting analysis through group mean centering, which is also known as the Frog-Pond model. In cases of 0.77<E<1.3, where between and within group variations are similar, individual-level analysis is recommended [5,8-10].

(1) $$E\;=\;\frac{\eta_{B}}{\eta_{w}}\;\;\;$$

WABA(2) differs from WABA(1) in that it not only uses the η values, but also considers the correlation between independent and dependent variables. The formula for WABA(2) is as follows:

(2) Total correlation=between component + within component                                  

(3) $$r_{\mathrm{XY}}\;=\;\eta_{\mathrm{BX}}\eta_{\mathrm{BY}}\eta_{\mathrm{BXY}}\;+\;\eta_{\mathrm{WX}}\eta_{\mathrm{WY}}\eta_{\mathrm{WXY}}\;\;$$

η_BX_ =between-unit η value for independent variable
η_BY_ =between-unit η value for dependent variable
r_BXY_ =between-unit correlation coefficient between independent and dependent variables
η_WX_ =within-unit η value for independent variable
η_WY_ =within-unit η value for dependent variable
r_WXY_ =within-unit correlation coefficient between independent and dependent variables

If between component (BC) value is larger than within component (WC) value, between-unit analysis is suitable, while within-unit analysis is appropriate if the BC and WC values are the same. A within-unit comparative analysis model needs to be established if the BC value is smaller than the WC value. Comparative analysis model involves manipulated variables from individual variables through cluster mean centering.

Lastly, statistical significance level was set to 0.05, and multivariate regression analysis was performed as per the WABA results; subsequently, the multi-level analysis was performed for purposes of comparison.

## RESULTS

### Basic characteristics of the subjects and community variables

Table 1 outlines basic characteristics of 215,737 subjects involved in the analysis. There were more females than males, and self-rated health scores were somewhat higher in males (3.31) than in females (3.06). The respondents aged 19-45 were 34.8% of the total, the other 37.5% were 46 to 65, and the remaining 27.7% were above 65. Self-rated health scores gradually decreased with aging. In addition, lower education level and income showed correspondingly lower self-rated health score. Majority of the subjects were married (68.5%), but single subjects had highest self-rated health score (3.65). Increasing contact frequency to neighbors was correlated with increased self-rated health score, but there was no correlation observed between contact frequency with family or friends and self-rated health score.

Table 2 outlines the results of assessing correlation between the individual variables. Regional level qualitative SC and gap SC – gap between regional level SC and individual level SC – had a very high correlation at 0.88. Contact frequency with family, neighbors, or friends and individual level qualitative SC had low correlation, indicating the possibility of concurrent introduction with other sociodemographical or social-economical control variables.

### Within and between analysis

To assess the validity of selected variables from community residents’ average responses of qualitative SC, a WABA was performed. From the WABA(1) analysis of regional level qualitative SC variables, η_B_ value was 0.219 and η_W_ value was 0.781. E value calculated according to the equation (1) is 0.280, and fits into E≤0.77 group with E (15°) as a standard, indicating large modification within group and the consequent adequacy of analysis using single-level comparative model.

Next, according to the analysis using the WABA(2) equation, BC was 0.005, and WC was 0.072. Since WC score was higher than BC score, similar to the suggestion from WABA(1), the analysis using the single-level comparative model is the appropriate method.

Based on the WABA results, a single-level analysis was performed and an additional multivariate regression analysis using cluster mean centered individual variables was performed as a comparative model (Table 3).

### Single- and Multi-level regression analysis

WABA results indicated that analysis of qualitative SC between groups was not appropriate, and analysis using comparative model was considered. A new variable was generated by cluster mean centering of qualitative SC for further analysis. Increased cluster mean centered qualitative SC was correlated with increased self-rated health score (0.054, p<0.001). It is notable that this result was not very different from the result of model 1 which involved qualitative SC, which was not cluster mean centered.

The comparisons between model 1, model 3, and model 4 provide results for unconditional multi-level analysis and multi-level analysis reflecting every variable. Unconditional model 3 without variable introduction had intra coefficient correlation (ICC) value of 0.031, and model 4 which had all variables introduced showed statistical significance in upper-level variable of regional average value for individual qualitative SC (0.068, p<0.001) with ICC value of 0.01 (Table 4).

## DISCUSSION

Since the 2000s, health-related studies using multi-level analysis are on the rise; however, there is still no clear standard for statistical test procedures prior to the analysis, unlike single-level multivariate regression analysis. Only a few studies provide preliminary validation procedures to show regional differences causing different characteristics of dependent variables or to assess validity in the manipulation of individual responses into community variables. In other words, there is possibility of artificial analysis results due to unfulfilled statistical requirements, even if the multi-level analysis model is appropriate in theory. The WABA can partially solve these level-issue problems. Therefore, this study performed statistical validation using WABA to assess the validity of merged and averaged community variables calculated from the community residents’ responses of reliability and reciprocity variables – measuring qualitative aspect of SC – in 2013 Community Health Survey, and the possibility of utilizing the result in multi-level analysis.

From the two analysis methods suggested by WABA, both WABA(1) – utilizing individual variables to assess within group variance and between group variance – and WABA(2) – considering correlation between independent and dependent variables and within group variance and between group variance of these variables – results demonstrated appropriateness of using the single-level comparative model.

Based on the above result, single-level multivariate regression analysis was performed using cluster mean centered qualitative SC, calculated by subtracting merged and averaged community scores from individual scores. There was a statistically significant association between the cluster mean centered qualitative SC and self-rated health. However, these results—compared to the values from merging individual-level reliability and reciprocity scores—are neither very high nor significantly affect the coefficient value. Since the correlation between cluster mean centered qualitative SC and simple merged qualitative SC is very high (0.88), introduction of any variable would yield similar results. In this analysis, manipulation of two “yes or no question” about regional awareness in to continuous variable resulted in a very limited score range. Therefore, it appears that statistical significance was achieved even though the meanings between the two variables are practically different. Other factors such as love for the community, sense of belonging, and trust level in policies—aside from reliability and reciprocity, which were assessed in this study —are important [11], but these psychological characteristics were not reflected properly. Therefore, using a validating tool to assess regional qualitative SC, or re-designing the survey to reflect Korean culture and better examine and compare regional qualitative SC and SC differences between communities, can also be considered.

Moreover, to compare multi-level analysis results, we looked at the variance size in unconditional model, and it was statistically significant at 0.03, exhibiting community-level variance although at low level. However, ICC analysis involving measurement of community-level variance from the whole variance indicated variance due to regional characteristics is only at 0.031 (3.1%). Typically, a multi-level analysis is not performed if the ICC analysis result is less than 5.0% as between group homogeneity is not secured [12], and community-level variance can change by introduction of individual-level control variables 3.1% from the null model, have no independent variables and only set outcome variable, is a comparatively small value. On the other hand, multi-level analysis with all variables introduced showed correlation between self-rated health score and regional qualitative SC, but there was not much difference in the averaged qualitative SC coefficient value from the single-level model. There is a possibility that significance observed in regional variables from multi-level analysis is a statistical artifact, looking at within group variance and the size of between group variance. In conclusion, the single-level comparative model suggested by both the WABA(1) and WABA(2) test result is suitable for the analysis.

Meanwhile, external factors for inadequacy of multi-level analysis for WABA using self-rated health and qualitative SC include potential lack of difference in self-rated health among cities, districts, or boroughs in Korean society. This is due to frequent moving and shortened time periods for stable housing; furthermore, the majority of interactions performed in communities or different regions is outside the house [14]. Even if a more accurate method is used to assess self-rated health as a variable, since it is unlikely to be explained by community variables, a multi-level analysis is practically not feasible. However, some domestic multi-level analysis studies indicate an association between regional characteristics and health [14-17]. This might imply that people having high sensitivity to exposures at the community level is more reactive with qualitative SC of regional level for the analysis, rather than there are only small differences on health-impacting community-level qualitative SC among the communities; therefore, the study hereafter need to be performed on a selected cohort of samples based on high-sensitive to the environment. In other words, as the value from the WABA(2) analysis reflect the correlation between independent and dependent variables, a follow-up study introducing highly-correlated health variable is needed.

There are still a few limitations in the effective usage of the WABA as an analytical tool to test whether or not multi-level analysis can be performed. First, the WABA is an analysis-of-variance based method, and only works when the dependent variable is a continuous variable. Therefore, dichotomous variables such as disease morbidity or health behaviors limit the WABA. Moreover, according to previous studies, when Between variance and Within variance values are exactly same in WABA(2) analysis, individual single-level analysis is appropriate. However, the two values being identical is an extremely rare case; therefore, the chance of single-level analysis being concluded as an appropriate method is very low. These limitations of the WABA indicate that when performing multi-level analysis, validation using WABA is a sufficient but not a necessary condition. Nevertheless, WABA is a useful analytical tool, when manipulating continuous variable that can represent a community into community-level variables and assessing its utility in multi-level analysis.

This study is a first attempt—within the scope of our investigation—to check if the manipulation of SC variable from individual responses into community variable is appropriate, and also if the manipulated variable is adequate in multi-level analysis, using WABA. Although our research showed that qualitative SC was deemed inappropriate for multi-level analysis due to large within group variance, validity testing should be performed using the WABA as a statistical tool in future multi-level health studies involving merged and averaged data.

## Figures and Tables

**Table 1.** Basic characteristics of the subjects

Characteristic	n	%	Average± standard deviation
Sex			
Male	96,721	44.8	3.31 ±0.93
Female	119,006	55.2	3.06±0.95
Age (yr)			
19-45	75,121	34.8	3.56±0.76
46-65	80,957	37.5	3.23±0.86
≥65	59,649	27.7	2.59±0.99
Education			
None schooling	26,833	12.4	2.38±0.96
Elementary	35,032	16.2	2.77±0.95
Middle	24,145	11.2	3.07±0.89
High	71,584	33.2	3.41 ±0.84
Above college	58,133	26.9	3.54±0.76
Household monthly income (million Korean won)			
<1	52,884	24.5	2.65 ±1.01
1-2	39,022	18.1	3.16±0.92
2-3	39,477	18.3	3.35±0.84
3-4	25,044	11.6	3.42±0.81
>4	52,800	24.5	3.47±0.81
No response	6,500	3.0	3.13±0.95
Marital status			
None married	36,916	17.1	3.65±0.82
Divorced, bereaved	30,961	14.4	2.72±0.82
Married	147,850	68.5	3.19±0.90
Neighborhood contact			
Rare	80,144	37.2	3.00±0.99
Occasional	74,919	34.7	3.23±0.93
Frequent	60,664	28.1	3.33±0.88
Friend contact			
Rare	51,534	23.9	3.28±0.95
Occasional	118,495	54.9	3.28±0.87
Frequent	45,698	21.2	2.79±1.02
Family contact			
Rare	46,076	21.4	3.15±0.96
Occasional	131,510	61.0	3.19±0.93
Frequent	38,141	17.7	3.15±0.99

**Table 2.** Correlation between variables

	(1)	(2)	(3)	(4)	(5)	(6)	(7)	(8)	(9)	(10)	(11)
(1) Self rated health	1	-0.13^***^	-0.39^***^	0.39^***^	0.29^***^	-0.07^***^	0.00	0.14^***^	-0.16^***^	-0.10^***^	-0.05^***^
(2) Sex		1	0.04^***^	-0.18^***^	-0.05^***^	0.14^***^	-0.08^***^	-0.10^***^	0.06^***^	0.01	0.01
(3) Age			1	-0.67^***^	-0.44^***^	0.03^***^	-0.05^***^	-0.38^***^	0.14^***^	0.36^***^	0.25^***^
(4) Education				1	0.49^***^	-0.15^***^	0.03^***^	0.39^***^	-0.14^***^	-0.34^***^	-0.20^***^
(5) Income					1	-0.19^***^	0.01^***^	0.25^***^	-0.11^***^	-0.20^***^	-0.08^***^
(6) Marriage						1	0.09^***^	0.06^***^	-0.03^**^	-0.06^***^	-0.07^***^
(7) Family contact							1	0.19^***^	0.11^***^	-0.11^***^	-0.06^***^
(8) Neighborhood contact								1	0.05^***^	-0.51^***^	-0.35^***^
(9) Friend contact									1	0.00	-0.01
(10) Qualitative SC										1	0.88^***^
(11) Gap SC^1^											1

SC, social capital.

1 Gap between regional level SC and individual level SC.

** p<0.01,

*** p<0.001.

**Table 3.** Within group and between group variance analysis

Variable	Correlation	Etas		Correlation		Components		Result
		Between	Within	Between	Within	Between	Within	
Self rated health		0.032	0.968					
Qualitative social capital	0.724	0.219	0.781	0.756	0.095	0.005	0.072	BC< WC parts

BC, between component; WC, within component.

**Table 4.** Estimates of single-level and multi-level regression analysis

	Model 1	Model 2	Model 3	Model 4
Intercept	-3.044^***^	-2.917^***^	-2.825^***^	-3.020^***^
Sex				
Male	Reference	Reference		Reference
Female	-0.115^***^	-0.120^***^		-0.118^***^
Age (yr)				
19-45	Reference	Reference		Reference
45-64	-0.161^***^	-0.158^***^		-0.147^***^
Above 65	-0.455^***^	-0.451^***^		-0.437^***^
Education				
None schooling	Reference	Reference		Reference
Elementary	0.204^***^	0.199^***^		0.200^***^
Middle	0.355^***^	0.344^***^		0.350^***^
High	0.520^***^	0.504^***^		0.510^***^
Above college	0.578^***^	0.560^***^		0.570^***^
Household monthly income (million KRW)				
<1	Reference	Reference		Reference
1-2	0.171^***^	0.168^***^		0.174^***^
2-3	0.235^***^	0.231^***^		0.238^***^
3-4	0.254^***^	0.248^***^		0.260^***^
>4	0.288^***^	0.282^***^		0.296^***^
No response	0.165^***^	0.162^***^		0.167^***^
Marital status				
None married	Reference	Reference		Reference
Divorced, bereaved	0.180^***^	0.180^***^		0.177^***^
Married	-0.023^***^	-0.026^***^		-0.027^***^
Family contact frequency				
Frequent	Reference	Reference		Reference
Occasional	-0.001	0		0
Rare	-0.036^***^	-0.040^***^		-0.050^***^
Neighborhood contact frequency				
Frequent	Reference	Reference		Reference
Occasional	-0.036^***^	-0.050^***^		-0.046^***^
Rare	-0.072^***^	-0.106^***^		-0.104^***^
Family contact frequency				
Frequent	Reference	Reference		Reference
Occasional	-0.049^***^	-0.049^***^		-0.053^***^
Rare	-0.202^***^	-0.201^***^		-0.215^***^
Qualitative SC	0.071^***^	-		-
Centering SC	-	0.054^***^		-
Level 2				
Qualitative SC (regional)				0.068^***^
σ2 (level 1)			0.869^***^	0.681^***^
τ00 (level 2)			0.028^***^	0.007^***^
ICC			0.031	0.010

KRW, Korean won; SC, social capital; ICC, intra coefficient correlation.

^***^p<0.001.
