# Supplementary material for: Validity analysis on merged and averaged data using within and between analysis: focus on effect of qualitative social capital on self-rated health
Source: Epidemiol Health. 2016 Apr 8;38:e2016012. doi: 10.4178/epih.e2016012 (PMC4914543; doi:10.4178/epih.e2016012)
Supplement: Supplementary file 1 [file epih-38-e2016012-supplementary.pdf]

Title

A validity analysis on merged and averaged date using WABA

Running Title

- **Focused on Effect of qualitative social capital on Self rated Health**

First Author

Sang soo Shin, Department of Preventive Medicine, Hanyang University College of Medicine, 222 Wangsimni-ro, Sungdong-gu, Seoul 04763, Korea Tel: +82-2-2220-0668, Fax: +82-2-2293-0660, E-mail: bskyts@daum.net

- Affiliation : Department of Preventive Medicine, Hanyang University College of Medicine, Seoul, Korea

Corresponding

Young-jeon Shin Department of Preventive Medicine, Hanyang University College of Medicine, 222 Wangsimni-ro, Sungdong-gu, Seoul 04763, Korea Tel: +82-2-2220-0668, Fax: +82-2-2293-0660, E-mail: yshin@hanyang.ac.kr

Affiliation : Department of Preventive Medicine, Hanyang University College of Medicine, Seoul, Korea

초록

목적 : 건강결정요인으로 지역사회자본을 주목한 연구가 증가하면서, 일부 연구에서는 지역사회자본 자료 활용을 위하여 지역 주민 응답치의 합산 평균을 구하고, 이를 이용한 다수준 분석을 수행하고 있다. 합산 평균 데이터를 활용하기 위해서는 합산한 자료가 지역 특성으로 산출하는 것으로 타당한지, 다수준 분석에 활용하는 것이 적합한지에 대한 충분한 검토가 필요하다. 따라서 이 연구는 WABA 기법을 이용하여 산출된 지표의 타당성과, 다수준 분석의 적용가능성을 파악하고자 한다.

방법 : 2013년 지역사회건강조사를 활용하여 신뢰와 호혜성 문항의 지역 주민 응답치를 합산 평균한 자료를 지역변인으로 생성한 후, 주관적 건강감 변인을 종속 변인으로 한 WABA 분석을 실시하였다. 다음으로 WABA 분석 결과가 제안한 모형에 준하여 분석을 실시하였다.

결과 : WABA(1)의 E 검정결과와, WABA(2)의 분석 결과 모두, parts로 판정되어 지역평균 중심화(cluster mean centering)한 질적 사회자본 변인으로 단수준 분석을 실시해야 하는 것으로 나타났다. 이를 바탕으로 다변량회귀분석을 수행한 결과, 지역평균 중심화한 질적 사회자본은 주관적 건강감에 긍정적 영향을 미치는 것으로 나타났으나(0.054, P<0.001), 중심화 하지 않은 사회자본 변인으로 분석했을 경우와 비교하여 결과에 큰 차이는 없었다.

결론 : 질적 사회자본의 변이는 지역간 보다는 지역내에서 높았기 때문에, 개인의 주관적 건강감과의 관계 분석시 개인 수준으로 분석해야 하며, 지역 평균 중심화한 값으로 분석할 것을 확인하였다. 향후 WABA를 비롯하여 지역변인 활용 타당성 및 다수준 분석의 적용가능성을 확인 할 수 있는 다양한 검정이 분석에 반영되어야 할 것이다.

중심단어

지역사회건강조사, WABA, 주관적 건강감, 질적 사회자본

Community Health Survey, WABA(Within and Between Analysis), Self-Rated Health, Qualitative Social Capital

서론

건강의 결정요인으로서 개인을 둘러싼 환경의 영향력에 대한 관심이 증가하면서, 개인과 지역 수준에서의 건강 발생요인을 동시에 반영하여 살펴보는 다수준 분석 연구가 증가하고 있다. 최근에는 개인에 건강에 영향을 미치는 요인으로 사회자본이 주목받고 있다. 사회자본은 개인이 아닌 사회적 관계 속에서 형성되는 자본의 형태로서, 경제자본, 인적자본, 문화자본과 구별되는 개념이다[1]. 사회 자본을 정의하는 학자들이 시각이 다양하지만, 사회참여, 가족 관계의 질, 연락빈도, 신뢰, 호혜성 등 다양한 대인관계로부터 발현되는 양적 요소, 질적 요소의 사회자본의 특성을 연구에 반영하고 있다.

이미 상당한 연구에서 사회자본과 건강과의 관련성이 검증되었고, 이제는 지역 수준에 존재하는 사회자본 특성을 이용한 다수준 연구가 등장하고 있다. 사회 자본을 지역 특성으로 반영하여 다수준 분석에 사용하기 위하여 자료를 선택하는 방법에는 크게 두 가지가 있다. 첫 번째는 투표율[2]과 자원봉사 캠프의 개수, 지역사회 기반 단체의 수[3]와 같은 행정자료를 사회자본의 대리변인으로 간주하여 분석에 반영하는 방식이다. 두 번째는 개인 응답치의 합산 평균 또는 응답율을 활용하는 방법이 있다. 지역사회 단체에 대한 평균 참여율[3], 지역사회 7개 집단에 대한 신뢰수준과, 지역사회 11개 유형의 조직에 대한 네트워크수준의 개인 응답치를 지역 변인으로 생성하고[4], 자신의 건강을 포함한 5개 영역에 대한 만족점수를 평균화한 값을 지역주민의 행복지수, 장애인을 비롯한 4개 영역에 대한 태도점수를 합산 평균한 값을 소수자에 대한 태도 점수를 사회자본의 속성으로 명명하여 분석에 반영하였다[5].

행정 자료를 대리 변인으로 활용하는 경우, 자료의 객관성을 확보할 수 있다는 장점을 가지고 있지만, 사회자본으로 인한 결과물인지, 사회자본 그 자체를 의미하는지 모호하다는 한계를 갖는다. 반대로 개인 자료를 합산하여 새롭게 생성한 자료는 행정 자료를 통해 산출한 변인에 비해 사회자본의 본질에 가까운 변인을 선정하는 것이 가능하지만, 설문대상자의 추출과정에서 편향이 발생할 경우, 합산자료의 신뢰성이 떨어질 가능성이 존재한다.

따라서 지역을 대표할 수 있도록 통계적으로 검증된 방식으로 추출한 설문 자료를 활용한다면 행정 자료를 통해서 산출 했을 때의 한계점을 보완할 수 있으며, 사회자본과 건강과의 관계를 설명을 위한 불필요한 비약을 방지 할 수 있다. 하지만 이러한 개인 응답치를 이용한 지역 변인화를 위해서는 통계적인 합산 타당성 검토가 요구된다. 개인점수의 결합으로 나타난 측정값이 그 집단의 특성을 적절히 반영하는지의 여부를 판단하기 위한 절차가 반드시 필요하다[6]. 다수준 연구의 구성 타당도 검증의 방법으로 유용한 절차로 그간 연구에서 제시하고 있는 방법론으로 WABA (Within And Between Analysis)가 있다. WABA는 자료합산의 정당성과, 다수준 분석의 적절성을 살펴 볼 수 있는 방법으로서, 새롭게 산출된 지역 변인 값이 정당하고 공유된 단위의 구성개념으로 사용할 수 있는지, 개인 변인으로 사용해야 하는지, 또는 비교 상황 변인으로 조작화 해야 하는지를 통계적 검정 결과치로 제시해 준다. 국내에서는 개인 변인을 활용하여 생성한 상위수준 변인의 타당도 검증 연구는 주로 조직학 연구에서 이루어졌으며[6][7][8], 국내보건의료분야

에서는 종속변인인 건강관련 삶의 질의 지역 요인 발굴을 위하여, 주관적 건강상태, 스트레스, 구강건강 변인을 합산 평균화한 변인들을 지역변인으로 활용하는 것이 타당한지를 WABA를 통해 검증한 이지혜 외의 연구(2013)가 유일하다[9]. 사회자본을 대상으로 한 합산 변인에 대한 타당성과 다수준 분석에의 적용가능성 검토는 이루어진 바 없다.

따라서 이번 연구는 2013년 지역사회 건강조사를 활용하여, 신뢰와 상호호혜성 변인을 합산 평균화 하여 산출한 지역 수준 변인에 대한 타당성 타당성을 검토하고, 주관적 건강감을 종속변수로 하는 다수준 분석에 적용 가능한지의 여부를 WABA 분석을 이용하여 검토하고자 한다.

대상 및 방법

1. 연구대상 및 자료

이번 분석에는 2013년 지역사회건강조사 자료를 활용하였다. 2013년 8월부터 11월까지 진행된 본 설문자료는 지역주민의 건강상태를 파악하여 근거에 기반한 보건정책을 수립 평가하기 위한 통계자료 생산을 위하여 매년 실시하고 있는 전국단위의 조사이다. 시군구 보건소 단위에서 표본층화 추출한 약 900여명의 19세 이상 성인을 대상으로 건강관련 문항을 설문하였으며, 전체 221,816명의 자료 중, 분석에 필요한 필수 문항에 응답하지 않은 6,089명을 제외한, 215,737명의 자료를 분석에 활용하였다.

2. 분석 변인

종속변인은 "평소에 본인의 건강은 어떻다고 생각합니까?"의 질문에 대하여, 매우 나쁨, 나쁨, 보통, 좋음, 매우 좋음으로 선택할 수 있도록 설계된 주관적 건강감 문항을 이용하였다. 주관적 건강감의 응답이 긍정적일수록 높은 점수를 주는 연속변인으로 활용하였다.

독립변수는 신뢰와 호혜성 여부를 활용하였다. 신뢰 문항은 "우리 동네 사람들은 서로 믿고 신뢰할 수 있다.", 호혜성 문항은 "이웃에 경조사가 있을 때, 주민 사이에 서로 도움을 주고받는 전통이 있다." 이며, 설문에는 예, 아니오 로 응답할 수 있도록 하였다. 여기에서 예 로 응답할 경우 1점, 아니오로 응답한 경우 0점으로 부여하고 두 문항의 응답치를 합산하여 0점에서 2점 사이의 분포를 갖도록 연속변인화 하였으며 이를 질적 사회자본으로 명명하였다. 또한 질적 사회자본의 지역 주민들의 개별 응답치를 합산 평균화하여 지역변인화 하였으며, 마지막으로 지역 합산 평균치를 개인 응답치에서 뺀, 즉 지역 평균 중심화(cluster group mean centering)한 변인을 산출한 후 이를 평균 중심화한 질적사회자본 변인이라 명명하였다.

통제변수는 성별, 연령, 학력, 소득, 혼인상태, 가족, 친구, 이웃과의 연락빈도로 구성하였다. 성별은 남, 녀, 연령은 19세 이상 45세 미만, 45세 이상 65세 미만, 65세 이상, 학력은 무학, 초졸, 중졸, 고졸, 초대졸 이상, 월 가구소득은 100만 미만, 100만 이상 200만 이하, 200만 이상 300만

이하, 400만 이상, 응답거부, 혼인상태는 미혼과, 기혼, 혼인 후 배우자 없음의 명목 변수로 구성하였다. 가족, 친구, 이웃과의 연락빈도는, 1주일에 4회 이상, 1주일에 3회 이하부터 한달에 한번 이상, 한달에 1번 미만의 연락빈도 응답 변인에 대하여 각각 '빈번한', '가끔', '드문'의 명목변수로 재구성 하였다.

3. 통계분석

먼저, 자료의 기본 특성을 확인하기 위하여 분석에 사용되는 변인들의 기초 통계량을 산출하였다. 다음으로 신뢰와 호혜성 변인을 합산하고, 지역 주민의 응답 평균을 지역변인으로 새롭게 생성한 후 통제변인과 독립변인의 Spearman 상관관계를 분석하였다. 이어서 WABA를 이용하여 타당성 분석과, 적합도 분석을 실시하였다. WABA는 합산의 적절성을 검증할 뿐 아니라 변인 간의 관계를 바탕으로 분석 수준을 제시하는데, WABA(1)과 WABA(2)으로 나누어 분석을 수행할 수 있다. WABA(1)은 각 독립 변인에 대하여 개별적으로 분산분석을 실시한 결과를 이용하여, 지역 변인의 타당성 여부를 보여주고, WABA(2)는 독립 변인과 종속 변인간 관계에 대하여 집단 간 차이와 집단 내 차이를 이용하여 분석 수준을 결정할 수 있게 한다[6][9][10].

WABA(1)는 집단내 분산과 집단간 분산의 크기를 고려하는 방법으로서 η_B/η_W 값으로 검정한다. 에타(η_w)은 SSW/SST의 제곱근, 에타(η_B)은 SSB/SST의 제곱근으로 산출하고, 그 결과 값을 E-검정한다. E-검정은 두 벡터(B와 W)는 90°를 형성하는 두 개 축 내에서 집단간 편차 값과 집단 내 편차값이 움직이는데, 벡터의 중간인 45°를 중심으로 7.5°씩 떨어진 52.5°일때의 E값인 1.3과 37.5°일때의 E값인 0.77을 검정 기준으로 한다. E≥1.3인 경우 집단간 차이가 있고, 집단 내 동질성이 있는 것으로 판단할 수 있고, E≤0.77인 경우 집단 내 차이가 크고, 상대적으로 집단간 차이가 없으므로 개인 수준 변인에 집단 평균 중심화 하여 분석할 것을 제안하고 있으며, 이를 비교(Frog-Pond)모형이라고 표현한다. 0.77<E<1.3은 집단간, 집단내 분산이 비슷하므로 개인단위로 분석할 것으로 제안한다[6][9][10][11].

$$E = \frac{\eta b}{\eta w} \quad \text{.................... (1)}$$

WABA(2)는 에타 값을 이용할 뿐만 아니라 종속변인과 독립변인의 상관관계를 감안하여 산출한다는 점에서 WABA(1)과 다르며, 산출 공식은 다음과 같다.

Total correlation = between component(BC) + within component(WC) .... (2)

$$r_{XY} = \eta_{BX}\eta_{BY}r_{BXY} + \eta_{WX}\eta_{WY}r_{WXY} \quad \text{.................. (3)}$$

여기에서 BC가 WC보다 크면 집단수준으로 분석이 적합하고, BC가 WC와 같으면 개인수준 분석,

BC가 WC보다 작으면 개인 수준 비교 모형으로 구축할 것으로 판정한다. 비교모형 분석은 집단 평균 중심화하여 개인 변인을 조정하는 것을 의미한다.

마지막으로 통계적 유의 수준은 0.05로 하여, WABA 결과가 제시하는 분석 방향에 맞추어 다변량 회귀분석을 실시하였다.

분석 결과

1. 대상자 특성 및 지역변인의 기초 특성

분석 대상에 포함된 215,737명의 주요 기초특성은 [Table 1]과 같다. 여성이 남성보다 응답자 수가 많았으며, 남성의 주관적 건강감이 3.31점으로 여성의 3.06점에 비해 다소 높았다. 19세부터 45세 사이의 연령집단이 전체 조사자의 37.5%를 차지하였으며, 46세 이상 65세 미만은 37.5%, 65세 이상이 27.7%였다. 연령집단이 증가할수록 주관적 건강감 점수는 감소하였다. 학력의 경우 저학력 집단일수록, 소득의 경우 저소득일수록 주관적 건강감 점수가 낮았다. 혼인여부의 경우 혼인상태의 참여자가 68.5%로 가장 높았으며, 미혼이 3.65점으로 점수가 가장 높았다. 이웃과의 연락빈도는 이웃과의 연락빈도가 증가할수록 주관적 건강감 점수도 증가하는 것으로 나타났으나, 가족과 친구 연락빈도 증가와 주관적 건강감 점수는 일관적 경향이 나타나지 않았다.

[Table 1] 분석대상의 기초특성

개인 변인간 상관관계를 분석한 결과는 [Table 2]와 같다. 지역수준의 질적 사회자본과, 개인수준의 질적 사회자본의 격차로 산출한 변인과, 개인수준의 질적 사회자본의 상관관계가 0.88로 매우 높은 것으로 나타났다. 가족, 이웃, 친구와의 연락빈도와 개인수준 질적 사회자본과의 상관관계는 높지 않아, 다른 인구사회학적, 사회경제적 통제변인들과 함께 투입 가능한 것으로 나타났다.

[Table2] Correlation between Variables

2. WABA 분석 결과

지역 주민의 질적 사회자본의 응답 평균치로 산출한 변인의 정당성을 파악하기 위하여, WABA 분석을 실시하였다. 지역수준의 질적 사회자본 변인의 WABA(1)을 분석한 결과, η_B 는0.219 η_W 값은 0.781로 나타났다. 이를 (1)의 공식에 따라 계산하면, E 값이 0.280으로 나타나, E(15°) 기준으로

0.77이하에 해당하여 집단 내 변이가 크므로, 단수준 비교 모형으로 한 분석이 적합함을 알 수 있다.

다음으로 WABA(2)의 공식을 이용하여 분석한 결과 Between Components는 0.0053, Within Components는 0.0719였다. 따라서 Within Components가 Between Components보다 높아, WABA(1)과 마찬가지로 단수준 비교 모형을 이용한 분석이 적절하다는 결론이 나타났다.

단수준 비교 모형을 이용한 분석이 필요하다는 검정 결과를 바탕으로 단수준 분석을 실시하되, 비교 모형으로서 지역 평균 중심화한 개인변인으로 다변량 회귀분석을 실시하였다.

[Table 3] Result of Within and Between variance analysis

3. 다변량 회귀분석 결과

WABA의 분석 결과, 질적 사회자본을 집단 수준으로 분석하는 것이 적절하지 않으며, 비교 상황 모형으로 분석해야 할 것을 제안하고 있다. 이를 위하여 개인의 사회자본을 지역 평균의 질적 사회자본 수준을 지역 평균 중심화(Cluster mean centering)하여 새롭게 생성한 변수로 분석을 실시하였다. 그 결과 지역평균 중심화한 질적 사회자본의 점수가 증가할수록 주관적 건강감이 증가하는 것으로 나타났다(0.054, $p<0.001$). 다만 이 결과는 평균 중심화하지 않은 질적 사회자본을 투입한 Model 1의 결과와 비교하여 크게 차이 나지 않는 결과였다.

[Table 4] 다변량 회귀 분석 결과

고찰 및 결론

2000년대 이후 다수준 분석 기법을 이용한 건강 연구가 증가하고 있지만, 단수준 다변량 회귀분석과 달리 다수준 분석을 수행하기 전에 점검해야 할 통계 검정 절차에 대한 명확한 기준이 없는 상황이다. 이에 따라 대부분의 연구에서는 분석을 위한 선행 점검 없이 이루어지고 있다. 이론적으로는 다수준 분석으로의 설계가 타당하더라도, 통계적으로는 적합하지 않을 수 있는데, WABA는 이러한 레벨이슈에 문제를 부분적으로 해결해 준다. 따라서 이 연구는 2013년 지역사회 건강조사 중 사회자본의 질적 측면을 측정한 신뢰와 호혜성 변인의 지역 주민 응답치를 합산 평균하여 지역변인화 하였을 때, 새롭게 생성한 변인이 타당성을 갖고, 다수준 분석에서 활용 가능한지를 살펴보기 위하여 WABA 분석을 이용하여 통계적 검증을 실시하였다.

WABA에서 제시한 두 가지 측정방식으로 분석한 결과, 독립 변인만을 활용하여 집단내 분산과 집단간 분산을 이용한 WABA(1)의 분석 결과에서는 단수준 비교 모형으로 측정하는 것이 적절하다는 결과가 나왔고, 독립변인과 종속변인의 상관관계와 이들 변수의 집단내 분산과 집단간 분산을 복합적으로 고려한 WABA(2)의 분석 결과에서도 단수준 비교 모형으로 설정하는 것이 타당하다는 분석 결과가 나타났다.

이를 바탕으로 개인의 사회자본을 지역 평균의 질적 사회자본 수준 값을 뺀, 즉 지역 평균 중심화한 질적 사회자본을 이용하여, 단수준 다변량 회귀분석을 실시하였다. 분석 결과, 평균화환 질적 사회자본 수준의 증가는 주관적 건강감의 향상에 통계적으로 유의한 관련성이 있는 것으로 확인되었다. 하지만 이러한 결과는 개인 수준의 신뢰와 호혜성 합산 값과 비교해서 특별히 유의수준이 높지 않았다.

주관적 건강감과 질적 사회자본을 이용한 WABA 분석의 검정 결과가 다수준 분석으로 적합하지 않은 원인으로는 첫 번째, 생성한 지역 변인이 지역간 격차를 잘 드러내기에 적합한 문항이 아닐 가능성이 있다. 현재 분석에 사용한 문항은 지역 인식에 대한 동의여부를 묻는 문항 2개를 연속변인화 하였기 때문에 지역의 질적 사회자본의 수준을 명료하게 드러낸다고 보기는 어렵다. 질적 사회자본은 신뢰나 호혜성 외에도, 지역사회에 대한 애착, 소속감, 제도에 대한 신뢰 도 중요한 요소이지만[12] 이러한 심리적 특성이 복합적으로 반영되지 못했다. 따라서 지역의 질적 사회자본을 면밀히 검정할 수 있는 검증된 설문도구를 활용하거나, 지역사회 사회자본을 잘 드러낼 수 있도록 한국적 맥락이 잘 반영된 문항으로 재 설계하여 지역간 사회자본의 격차를 살펴보는 방법을 검토할 수 있다. 두 번째, 실제 한국 사회는 시군구 행정 경계를 범주로 하는 지역간에는 질적 사회자본의 격차가 존재하지 않을 가능성이 있다. 주거지 이동이 잦고, 주거 기간이 짧으며, 주거지 보다는 다른 지역에서 또는 공동체를 중심으로 하는 상호작용이 이루어지기 때문이다[13]. 이러한 경우, 다른 사회자본 설문도구를 적용한다고 하여도 지역요인에 의한 설명 가능성이 낮기 때문에 다수준 분석이 현실적으로 불가능하다. 하지만 일부 국내 다수준 건강 연구에서 지역 특성이 건강과의 관련성을 보이는 결과들을 비추어 볼 때[14][15][16][17], 건강에 영향을 주는 지역 수준의 질적 사회자본의 지역간 변이가 작다기 보다는, 지역 수준의 질적 사회자본에 영향을 받을만한 질병 또는 건강행태를 선별하거나 인구 집단을 제한하여 분석하는 방법이 필요할 것이다. 즉, WABA(2)의 검증 값은 독립변인과 종속변인의 상관관계가 반영되기 때문에, 높은 수준의 상관성을 가지는 건강 변인을 투입한 후속 연구가 필요하다.

WABA가 다수준 분석의 수행 가능 여부를 판별하는 유용한 분석기법이라고 하기에는 여전히 몇 가지 한계점 또한 가지고 있다. 먼저 WABA는 기본적으로 분산 분석을 바탕으로 하는 분석 방법론이므로 종속변인이 연속변인일 경우에만 활용이 가능하다. 따라서 질병 이환 여부나, 건강 행태 여부와 같이 이분 변인으로 된 경우에는 여전히 WABA 분석을 하는데 제한이 따른다. 또한 기존 문헌에 따르면 WABA(2)분석에서 Between variance와 Within variance의 값이 같을 경우 이는

개인 수준인 단수준 분석이 적합하다는 판정이 내려지는데, 두 variance의 값이 완전히 동일한 경우는 극히 드물다. 따라서 단수준 분석이라는 판정 가능성은 상당히 희박하다. 이러한 WABA 가 가지고 있는 한계점은 WABA를 이용한 검증절차가 다수준 분석 수행을 위한 충분조건이지 필요조건이 아님을 보여준다. 그럼에도 불구하고, 지역적 대표성을 확보한 연속변인을 이용하여 지역 수준 변인으로 전환하고, 다수준 분석으로 활용가능한지를 검토할 때, WABA는 유용한 분석방법론임을 보여준다.

이 연구는 연구자가 확인한 범위 내에서, WABA 분석을 통해 개인이 응답한 사회자본 변인을 지역 변인화 하는 것이 타당한지, 그리고 생성한 변인이 다수준 분석에 적합한 것인지를 동시에 검토하였던 최초의 시도라는 의의를 갖는다. 비록 이 연구에서는 질적 사회자본이 집단내 변이가 크기 때문에 다수준 분석에 적합하지 않은 것으로 확인되었지만, 합산 평균화한 집단 자료를 이용한 다수준 건강연구를 수행할 경우, 이번 연구와 같이 WABA를 통계적 검정 수단을 이용한 타당성 검증의 절차가 필요할 것이다.

REFERENCE

1. Kim HY, A study of the effects of social capital on life satisfaction of the residents participating in mobilization program of community resources. J Korean Social Welfare Administration 2011;13(3):1-29 (Korean).
2. Kim CS, Han SY, Kim CW. The relationship between regional socioeconomic position and oral health behavior: A multilevel approach analysis. J Korean Acad Oral Health 2013;37(4):208-215 (Korean).
3. Jung MS, Cho BH. A Multi-level study of contextual effects of community capacity on health status among Seoul residents: Focused on social quality. Korean J Health Education and Promotion 2011;28(4):1-14 (Korean).
4. Kim HY. Community inequalities in health: The contextual effect of social capital Korean J Sociology 2010;44(2):59-92 (Korean).
5. Kim YH, Cho YT. Impact of area characteristics on the health of vulnerable populations in Seoul Korean J Population Study 2008;31(1):5-30 (Korean).
6. Park WW, Ko SK. Procedures and methods of multilevel analysis: with a focus on WABA Korean j management 2005;39(1):59-90 (Korean).
7. Han TY, Tak JK. Transformational/Transactional Leadership: Revisit using a multilevel approach Korean J Industrial and Organizational Psychol 2005;18(2):337-360 (Korean).
8. Lee YH, Lee KB, Lee SW. Individual perception or team perception? Application of multilevel analysis for securing validation of team level psychological evaluation Korean J sport psychol 2010;21(3):71-81 (Korean).
9. Lee JH, Lee DR, Kim SW, Lim DS, Heo TY. A study on the utilization of multilevel model using community health survey data J Korean data analysis society 15(3(B)):1289-1302

(Korean).

10. Klein KJ, Kozlowski SW. From micro to meso: critical steps in conceptualizing and conducting multilevel research Organizational Res Method 2000;3(3):211-236.
11. Dansereau F, Cho J, Yammarino FJ. Avoiding the "Fallacy of the wrong level" A within and between analysis(WABA) approach Group & Organization management 2006;31(5):536-577.
12. So, JK. Toward developing social capital indicators. J Korean regional development assoc 2004;16(1):89-117 (Korean).
13. Jung SW, Cho YT. Neighborhood characteristics and individual health under Korean context J Prev Med Public Heatlh 2005;38(3):259-266 (Korean)
14. Ko JE, Lee SH. A multilevel modeling of factors affecting depression among older Korean adults Mental health & social work 2012;40(1):322-351 (Korean).
15. Park YM, Kim YH. A spatially filtered multilevel model to account for spatial dependency: application to self-rated health status in South Korea Int J Health Geographics 2014;13(6).
16. Han SH. Compositional and contextual associations of social capital and self-rated health in Seoul, South Korea: A multilevel analysis of longitudinal evidence Soc Sci Med 2013;80:113-120.
17. Jung MS, Bigman-Galimore CA, Viswanath K. Contextual effects of community mobilization and communication capacity as a positive factor for self-rated health status: a multi-level analysis Int J Public Health 2014;59:289-299.
